# Leprosy Presenting With Scleroderma and Cataract: A Clinical Conundrum

**DOI:** 10.7759/cureus.64698

**Published:** 2024-07-16

**Authors:** Sana Ahmed, Maharshi Patel, Swarupa Chakole, Sonali Choudhari

**Affiliations:** 1 Community Medicine, Jawaharlal Nehru Medical College, Datta Meghe Institute of Higher Education and Research, Wardha, IND; 2 Medicine, Jawaharlal Nehru Medical College, Datta Meghe Institute of Higher Education and Research, Wardha, IND

**Keywords:** multidrug treatment, neuropathy, hypopigmentation, nlep, hansen’s disease

## Abstract

Leprosy, a chronic infectious disease caused by *Mycobacterium leprae*, even though treatable, remains a significant public health problem. It mainly impacts the skin, peripheral nerves, mucosa of the upper respiratory tract, and the eyes. In this case report, we present the case of a 64-year-old female with numerous hypopigmented patches with loss of sensations, madarosis, resorption of toes and digits, skin tightening, and diminution of vision. The skin over the hands exhibited thickening, leading to functional impairments that influenced both manual dexterity and mobility. The diagnosis of this unique case, showing a complex triad of lepromatous leprosy, scleroderma, and sclerotic cataract, was confirmed by clinical evaluation, skin biopsies, serological tests, and ophthalmic examination. Following this, the patient underwent dexamethasone-cyclophosphamide pulse therapy and multidrug treatment to halt the disease progression, prevent further disability, and reduce transmission. The case management addressed the issue of overlapping symptoms and conditions to provide appropriate care and cure to the patient. Public health initiatives under the National Leprosy Eradication Programme play an important role in promoting early diagnosis, effective treatment, and community empowerment, working toward a future where leprosy is no longer a threat to public health by preventing disability, reducing transmission, and combating the social stigma associated with it.

## Introduction

Leprosy, referred to as Hansen’s disease, is a debilitating, infectious disease primarily affecting the skin and peripheral nerves [1]. Leprosy, which manifests in a variety of clinicopathological forms contingent on the host's immunological status, is a serious problem for public health in developing countries, including India. Several clinical symptoms might range from a minor skin lesion to a widespread illness resulting in severe impairments and deformities [2]. The two polar clinical presentations of the condition are called "multibacillary" lepromatous leprosy and "paucibacillary" tuberculoid leprosy. There are many more common intermediate types with hybrid features [2]. Widely dispersed, symmetrically distributed skin macules, erythematous papules, nodules, and diffuse skin infiltration are typical of lepromatous leprosy. The disease mostly affects the face, ears, trunk, and limbs. Enlarged peripheral nerves are also commonly seen. Leprosy, the causative organism being *Mycobacterium leprae*, may be contracted through respiratory droplets from an untreated patient. The precise method of transmission is still unknown. The organisms are phagocytosed by lung macrophages upon inhalation of droplets, which subsequently spread throughout the circulatory system. Although cases have been observed affecting the muscles, eyes, bones, and other organs, it usually affects the skin and nerves since it only multiplies in tissues with relatively lower temperatures. The clinical spectrum of presentation might include anything from skin blemishes to severe illnesses that could cause abnormalities [3].

Autoimmune conditions such as scleroderma, characterized by fibrosis leading to skin thickening, present with a complex clinical spectrum ranging from limited cutaneous involvement to diffuse systemic manifestations impacting the patient’s quality of life and prognosis [4]. The sclerotic cataract, which causes opacities in the eye lens, represents rare but significant associations with leprosy. The pathogenesis of scleroderma involves a cascade of events leading to aberrant collagen deposition, vascular dysfunction, and immune activation within various tissues [4]. Leprosy remains a health concern in some parts of India. Data from the National Leprosy Eradication Programme reveal that 76% of new cases occur in Bihar, Maharashtra, Uttar Pradesh, Odisha, Chhattisgarh, Madhya Pradesh, West Bengal, and Jharkhand. This is true even if the nation was deemed "leprosy-free" in 2005 [5]. The World Health Organization (WHO) reports that 114,451 new leprosy cases were found nationwide in 2019-2020, making up 80.0% of cases in Southeast Asian nations [6]. According to WHO guidelines for the management of leprosy, a three-drug regimen of multidrug treatment (MDT) (Rifampicin, Dapsone, and Clofazimine) is recommended. The duration advised is six months for paucibacillary and 12 months for multibacillary leprosy [7]. Despite all efforts, there has been no discernible drop in the new case detection rate, a crucial statistical metric in leprosy control initiatives. To address the nation's public health needs and facilitate effective program planning and management, it is imperative to have a thorough understanding of the epidemiological profile. This case study presents the occurrence of leprosy in a patient concurrently diagnosed with scleroderma and sclerotic cataract, highlighting the complex interplay between infectious, autoimmune, and ophthalmic pathologies.

## Case presentation

A 64-year-old female homemaker presented with an eight-year history of numerous hypopigmented, poorly defined patches on the face, hands, feet, and trunk, with loss of sensations and difficulty swallowing. In addition, she complained of malaise and arthralgia. She also presented with a diminution of vision for four years.

**Figure 1:**
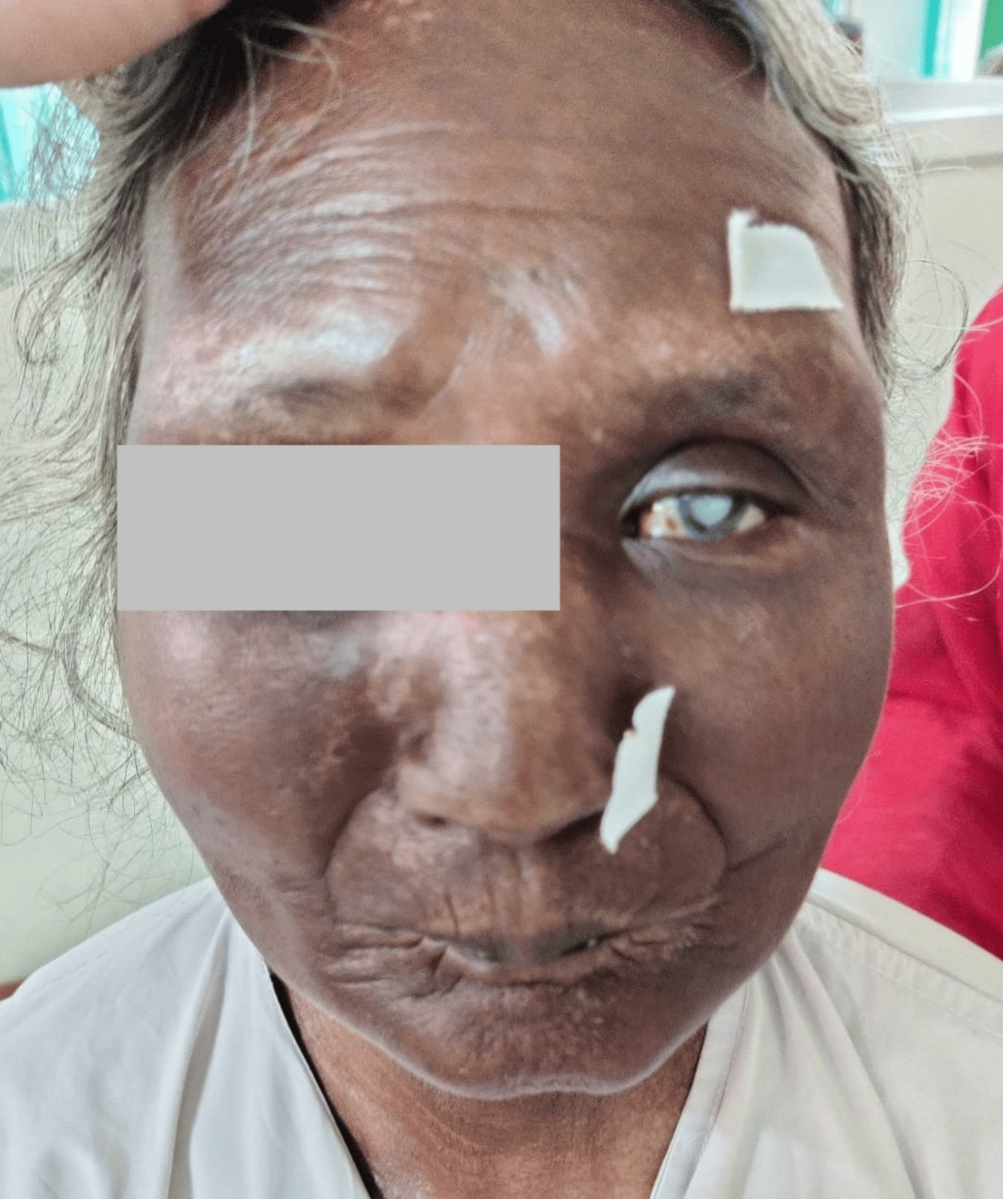
Loss of eyebrows and eyelashes, sclerotic cataract, purse-string-like appearance of the mouth, salt and pepper pigmentation

A general examination, post-consent, showed the patient to be conscious and well-oriented to time, place, and person. The patient was moderately built and nourished. The patient’s height and weight were 160 cm and 58 kg, respectively; the pulse was 86 beats per minute, regular; and the blood pressure was 118/74 mmHg in the supine position. There was no pallor, icterus, cyanosis, clubbing, or lymphadenopathy. The patient had numerous smooth, hypopigmented, and poorly defined skin lesions. There was a loss of eyebrows and eyelashes, the presence of a sclerotic cataract in the left eye, exposure keratopathy in the right eye due to lagophthalmos with a normal Bell's phenomenon, a purse-string-like appearance of the mouth, and a salt and pepper appearance of depigmentation on the face (Figure 1).

**Figure 2:**
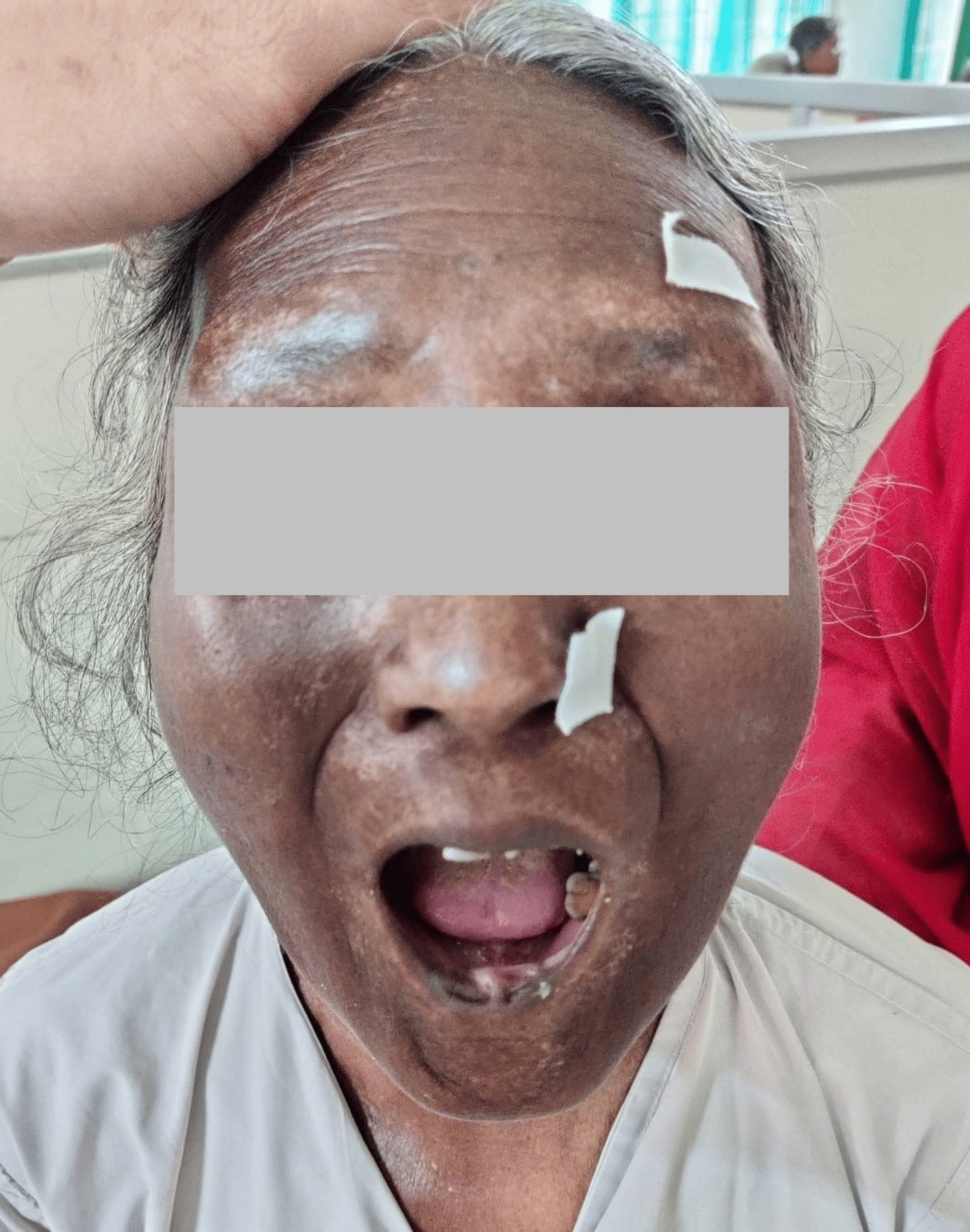
Multiple missing teeth

The nasal septum was depressed with no palate perforation. The patient was sensitive to touch on all mucosa without any palpable adenopathies. Her dental health was poor and revealed multiple missing teeth. Due to the low socioeconomic status, a proper dental care plan could not be followed (Figure 2).

**Figure 3:**
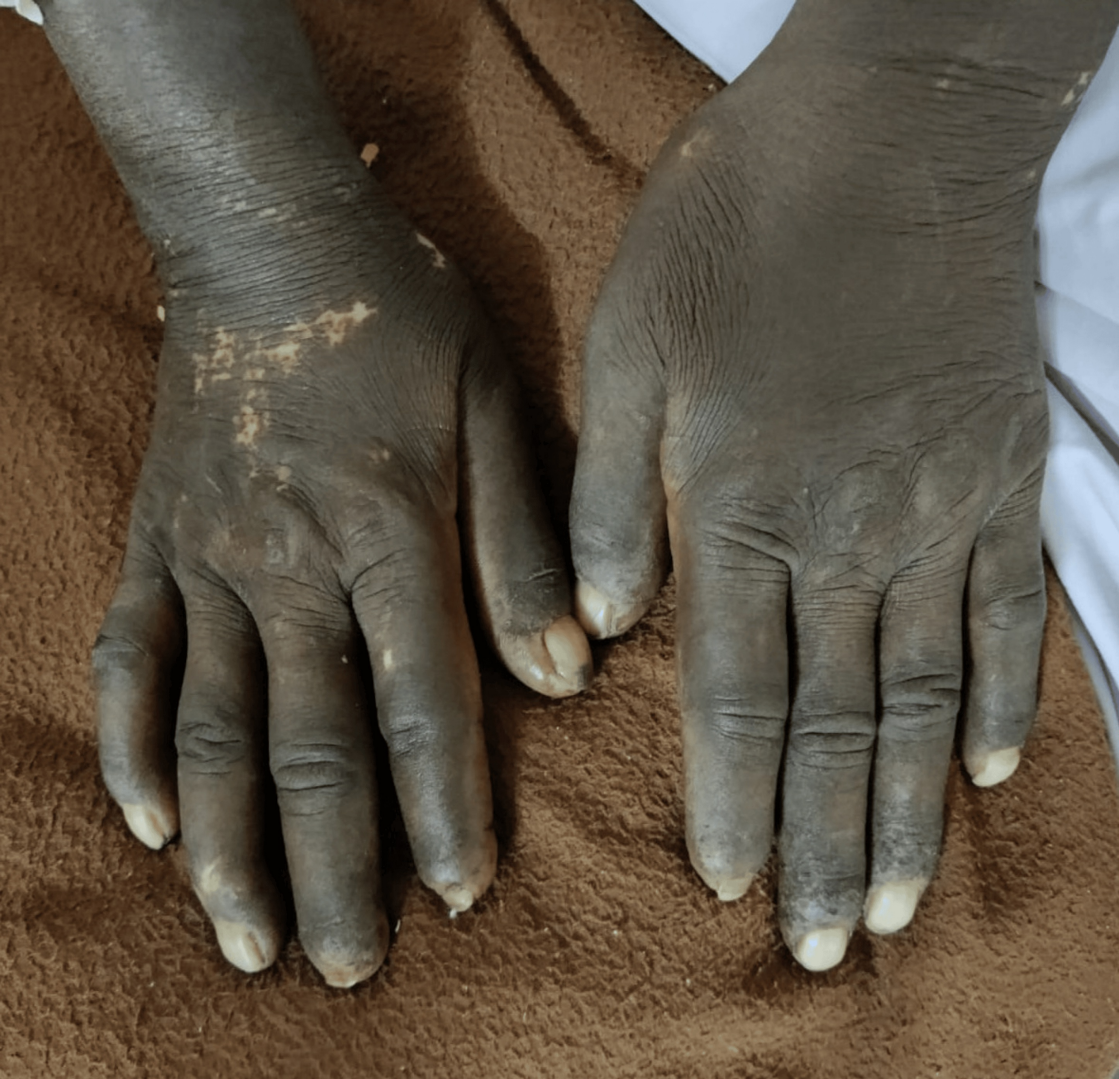
Skin tightening, ragged cuticles, resorption of digits

The skin over her hands was dry, hardened, and thickened, reminiscent of scleroderma, which led to functional impairment and deformities, impacting manual dexterity and mobility (Figure 3).

**Figure 4:**
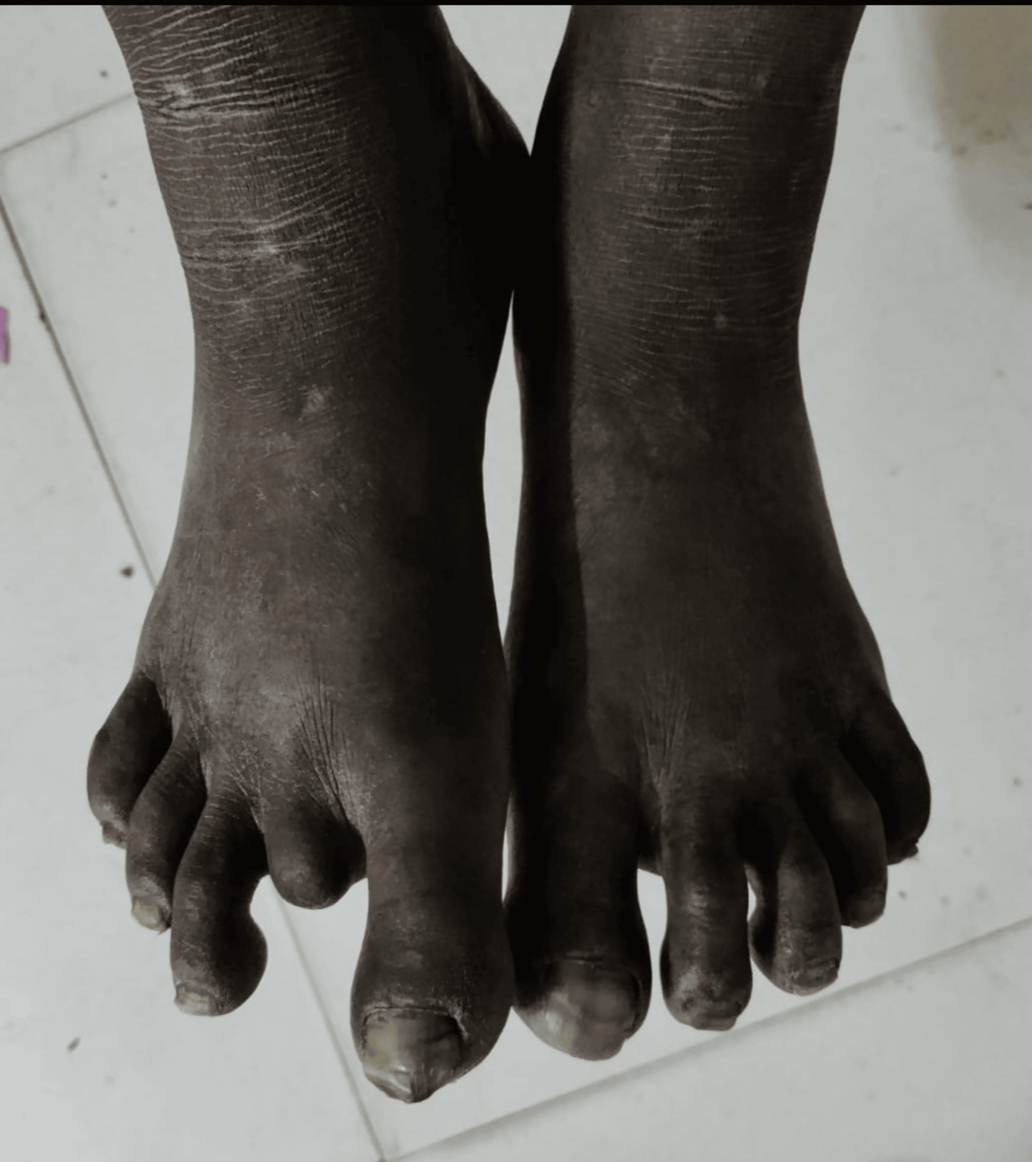
Resorption of toes

Resorption of toes was observed, indicating tissue loss and progressive deformities associated with advanced leprosy, necessitating multidisciplinary care and rehabilitation (Figure 4).

Ulnar and infraorbital bilateral nerve thickening were revealed during the peripheral nerve examination. The order of sensory loss in the presented case followed a distinctive pattern, with hot and cold differentiation being the most affected, followed by cold sensation, hot sensation, light touch, pain perception, and deep touch. This sequential deterioration of sensory functions provides valuable insights into the neurologic impact of leprosy. The patient exhibited glove and stocking anesthesia, indicating loss of sensations. The upper limbs elicited complete loss of sensations, while the lower limbs showed diminished sensations in a classic glove and stocking pattern. This finding aligns with the typical sensory distribution pattern observed in leprosy cases. Laboratory findings showed increased C-reactive protein levels, erythrocyte sedimentation rate, and a positive antinuclear antibody with the anti-centromere staining pattern (Table 1).

**Table 1:** Laboratory results of the patient

Investigations	Observed Value	Reference Value
Hemoglobin	11 g/dL	13-17 g/dL
Total leukocyte count	8000/mm^3^	4000-11000/mm^3^
Platelet count	180,000/mm^3^	150,000-400,000/mm^3^
Glucose	120 mg/dL	70-100 mg/dL
Sodium	138 mmol/L	137-145 mmol/L
Potassium	2.7 mmol/L	3.5-5.1 mmol/L
Albumin	3.6 g/dL	3.5-5 g/dL
Serum creatinine	0.5 mg/dL	0.5-1.2 mg/dL
Erythrocyte sedimentation rate	40 mm/hour	0-25 mm/hour
C-reactive protein	13.40 mg/L	<9 mg/L
Rheumatoid factor	Negative	<12 IU/mL
Antinuclear antibody	Positive, anti-centromere staining pattern	Less than 0.9 - not detectable; 0.9 to 1.1 - borderline positive; more than 1.1 - detectable (positive)

**Figure 5:**
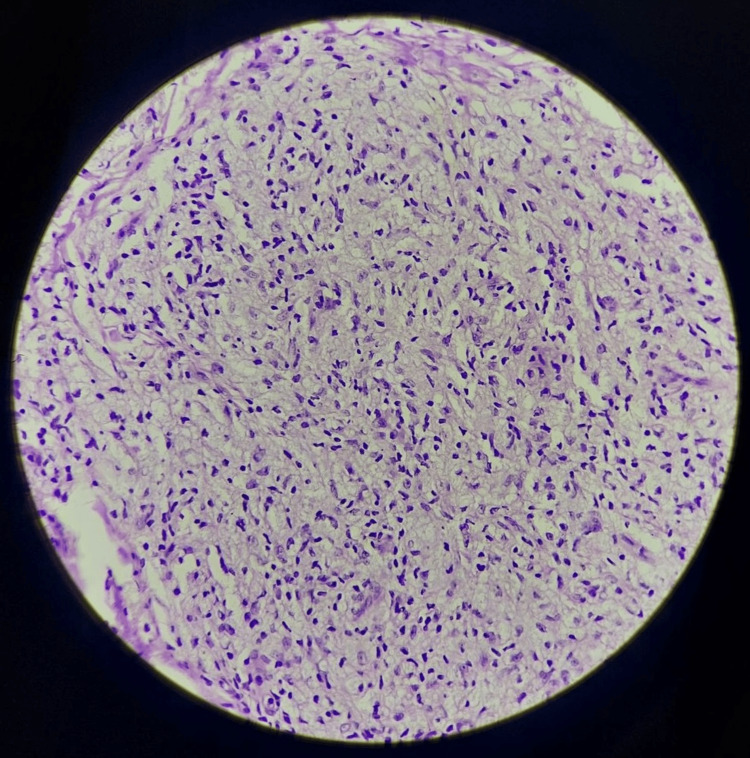
High-power image of hematoxylin and eosin stain showing a collection of foamy histiocytes forming a granuloma

On capillaroscopy, nail fold capillaries showed giant capillaries with micro hemorrhage and areas of capillary loss, consistent with scleroderma. Histopathology confirmed the diagnosis of lepromatous leprosy (Figure 5).

Clinical evaluations, skin biopsies, serological tests, and ophthalmic examinations confirmed the co-existence of leprosy, scleroderma, and sclerotic cataract. Management involved multidisciplinary care encompassing antimicrobial therapy, immunosuppressive treatment for scleroderma, and surgical intervention for cataracts. She was subsequently started on dexamethasone-cyclophosphamide pulse (DCP) therapy and MDT of Rifampicin 600 mg plus Clofazimine 300 mg monthly and Dapsone 100 mg plus Clofazimine 50 mg daily: 12 cycles in 18 months.

## Discussion

The socio-clinical case of lepromatous leprosy highlights its chronic, insidious nature and its co-existence with scleroderma and ocular manifestations. While the etiology of scleroderma remains multifactorial and not fully elucidated, genetic predisposition, environmental triggers, and immune dysregulation are believed to play significant roles [8]. Late diagnosis contributed to irreversible complications, including significant visual impairment and anatomical deformities, which cannot entirely be undone. The prevention of new impairments or the exacerbation of pre-existing impairments ought to be the primary objective. Assistive devices such as mittens and gloves made of soft cloth should be adopted to protect the hands from heat-related injuries while cooking. Adaptive devices, such as grip aids, can be customized to enhance dexterity [9]. Although these devices help improve the patient's functional ability, the extent of social participation, and the sense of independence, the stigma may be invoked when used outside their houses. Hence, proper counseling must be offered. The involvement of the peripheral nerves, skin, eyes, and musculoskeletal systems illustrates the systemic effects of the illness. The observed skin alterations not only exacerbate functional impairment but also pose significant challenges in differential diagnosis and therapeutic management.

*Mycobacterium leprae* is the cause of leprosy, one of the earliest diseases known to science, affecting humans [10]. It affects the eyes, upper respiratory tracts, and peripheral nerves. The case illustrates the early disease onset, the lengthy incubation period, and the prolonged use of Dapsone as a treatment. Thickening or hypopigmented lesions are common skin abnormalities associated with leprosy. The exact cause of the patient's illness is uncertain.

Comprehending the epidemiological profile is crucial for evaluating and addressing the nation's public health needs and facilitating efficient management and planning. The widespread incidence of leprosy has seen a dramatic decline since the adoption of MDT [11]. Analyses of current information revealed that 34 states and Union Territory (UT) had already reached the point of eradication, which is PR fewer than one case per 10,000 people. Between 2 and 5 prevalence rates per 10,000 people are still present in one state (Chhattisgarh) and one UT (Dadra and Nagar Haveli). There has been a modest increase in prevalence rates in five other states/UTs, namely, Odisha, Bihar, Chandigarh, Goa, and Lakshadweep, where eradication had previously been accomplished [11]. Examining the contacts of individuals afflicted with leprosy is a crucial approach, as it facilitates prompt identification, disrupts the chain of transmission, and averts deformities and impairments. This study is a thorough clinical assessment of people who currently have or previously had a leprosy diagnosis. The elderly and children are regarded as the special needs group. Notably, contact tracing ought to be carried out. It is advised to do a thorough initial assessment and ongoing, routine monitoring, both of which have not been done [12]. Lepromatous leprosy in teens and adults is confirmed to not always present with the well-known symptoms. Although the literature describes it as uncommon in this age group, it has been shown to occur very frequently. In low-endemicity areas, where healthcare professionals might be unfamiliar with the disease’s multiple clinical manifestations, leprosy nodules might play a crucial role [13].

Human rights and leprosy should be synonymous. These rights include freedom from cruel or humiliating treatment, equality before the law, and the right to live. Many countries have included these in their constitutions [14]. For those affected by leprosy, fair access to appropriate treatment and respect for human dignity are the most important human rights concerns. Public perceptions of people living with leprosy have improved in many areas because of the success of MDT in treating the disease and the recent wave of vigorous advocacy efforts. But in many nations, the stigma attached to leprosy has not entirely vanished. Discrimination against female leprosy patients is extremely severe in certain countries. The Laws and Regulations that could jeopardize the employment prospects of a person afflicted with leprosy, either domestically or abroad, should be repealed. Given that the greatest recognized risk factor for leprosy transmission is home transmission, surveillance techniques such as contact tracing are particularly crucial. Differentiating between leprosy lesions and other disorders can be challenging because of their comparable appearance, depending on the stage at which they are detected [15].

## Conclusions

The clinical manifestations of leprosy and the hypersensitivity reactions it causes can be strange. To prevent severe deformities and impairments and, more importantly, to stop the illness's dissemination, a thorough history and a high index of suspicion are still necessary for an early diagnosis and treatment. The National Leprosy Eradication Programme in India continues to make strides in combating leprosy. The multifaceted nature of scleroderma, characterized by its heterogeneous presentation, emphasizes the importance of a multidisciplinary approach to care. The National Scleroderma Foundation focuses on providing a range of resources and services aimed at improving the lives of those affected by it. All leprosy patients, including those who have been cured, should have a baseline ophthalmological examination. They should also be informed that to prevent avoidable blindness, prompt ophthalmological review is required for any new signs or symptoms. The rehabilitation of affected individuals is essential for treating more than just the physical symptoms; nevertheless, it also combats the social stigma and discrimination that often accompany the disease.
